# Comprehensive analysis of the interaction of antigen presentation during anti‐tumour immunity and establishment of AIDPS systems in ovarian cancer

**DOI:** 10.1111/jcmm.18309

**Published:** 2024-04-13

**Authors:** Wenhuizi Sun, Ping Xu, Kefei Gao, Wenqin Lian, Xiang Sun

**Affiliations:** ^1^ Department of Obstetrics and Gynecology, Guangzhou Women and Children's Medical Center Guangzhou Medical University Guangzhou China; ^2^ Department of Pathology, Guangzhou Women and Children's Medical Center Guangzhou Medical University Guangzhou China; ^3^ Department of Surgery, Guangzhou Women and Children's Medical Center Guangzhou Medical University Guangzhou China

**Keywords:** AIDPS systems, anti‐tumour immunity, bioinformatics, machine learning, ovarian cancer

## Abstract

There are hundreds of prognostic models for ovarian cancer. These genes are based on different gene classes, and there are many ways to construct the models. Therefore, this paper aims to build the most stable prognostic evaluation system known to date through 101 machine learning strategies. We combined 101 algorithm combinations with 10 machine learning algorithms to create antigen presentation‐associated genetic markers (AIDPS) with outstanding precision and steady performance. The inclusive set of algorithms comprises the elastic network (Enet), Ridge, stepwise Cox, Lasso, generalized enhanced regression model (GBM), random survival forest (RSF), supervised principal component (SuperPC), Cox partial least squares regression (plsRcox), survival support vector machine (Survival‐SVM). Then, in the train cohort, the prediction model was fitted using a leave‐one cross‐validation (LOOCV) technique, which involved 101 different possible combinations of prognostic genes. Seven validation data sets (GSE26193, GSE26712, GSE30161, GSE63885, GSE9891, GSE140082 and ICGC_OV_AU) were compared and analysed, and the C‐index was calculated. Finally, we collected 32 published ovarian cancer prognostic models (including mRNA and lncRNA). All data sets and prognostic models were subjected to a univariate Cox regression analysis, and the C‐index was calculated to demonstrate that the antigen presentation process should be the core criterion for evaluating ovarian cancer prognosis. In a univariate Cox regression analysis, 22 prognostic genes were identified based on the expression profiles of 283 genes involved in antigen presentation and the intersection of genes (*p* < 0.05). AIDPS were developed by our machine learning‐based integration method, which was applied to these 22 genes. One hundred and one prediction models are fitted using the LOOCV framework, and the C‐index is calculated for each model across all validation sets. Interestingly, RSF + Lasso was the best model overall since it had the greatest average C‐index and the highest C‐index of any combination of models tested on the validated data sets. In comparing external cohorts, we found that the C‐index correlated AIDPS method using the RSF + Lasso method in 101 prediction models was in contrast to other features. Notably, AIDPS outperformed the vast majority of models across all data sets. Antigen‐presenting anti‐tumour immune pathways can be used as a representative gene set of ovarian cancer to track the prognosis of patients with cancer. The antigen‐presenting model obtained by the RSF + Lasso method has the best C‐INDEX, which plays a key role in developing antigen‐presenting targeted drugs in ovarian cancer and improving the treatment outcome of patients.

## 1 INTRODUCTION

The World Health Organization ranks ovarian cancer as the fifth most lethal female cancer.^1^, ^2^, ^3^ One of the major challenges in treating ovarian cancer is the immunosuppressive tumour microenvironment (TME) that inhibits the anti‐tumour immune response. Antigen presentation is a critical process in activating T cells, and its dysregulation has been observed in various cancers, including ovarian cancer. MHC molecules, found on T lymphocytes and important for presenting antigens to them, can be downregulated in expression by tumour cells.^4^, ^5^ Because of this downregulation, tumour cells can avoid being attacked by the immune system. Transforming growth factor‐ (TGF‐) and interleukin‐10 (IL‐10) are two examples of immunosuppressive cytokines that can be secreted by tumour cells and further reduce antigen presentation.

Antigen presentation about tumours is essential for controlling the immune response to cancer.^6^, ^7^, ^8^, ^9^ The process involves the presentation of tumour‐associated antigens (TAAs) on the surface of tumour cells by major histocompatibility complex (MHC) molecules, which are then recognized by T cells. This interaction initiates a cascade of immune events, ultimately eliminating cancer cells and controlling tumour growth. This introduction will discuss the importance of antigen presentation in the tumour microenvironment and its implications for cancer immunotherapy. In preclinical studies, strategies to enhance antigen presentation in ovarian cancer have shown promising results. One potential strategy is the utilization of immune checkpoint inhibitors,^10^, ^11^, ^12^ which specifically target the programmed death‐1 (PD‐1) and programmed death‐ligand 1 (PD‐L1) pathways. Another strategy involves using tumour‐specific antigens in cancer vaccines, which can stimulate the immune system to target tumour cells. Additionally, adoptive T‐cell therapy (ACT) has shown potential in treating ovarian cancer by utilizing tumour‐infiltrating lymphocytes (TILs) or genetically modified T cells to target tumour‐specific antigens.

The immune system plays a pivotal role in monitoring and eliminating cancerous cells, but tumours have evolved multiple mechanisms to evade immune recognition.^13^ One such mechanism is impaired antigen presentation, contributing to immune tolerance towards tumour cells. However, recent advances in cancer immunotherapy have demonstrated the potential of harnessing the immune system to target and destroy tumours. Antigen presentation is a key component of this process, allowing the immune system to recognize and respond to tumour‐specific antigens. In this article, we will review the role of antigen presentation in tumours and discuss its potential applications in cancer immunotherapy. Several techniques regarding machine learning methods for constructing tumour prognostic models have been applied to this challenge. One popular approach is using supervised learning algorithms,^14^ which can be trained on data sets containing clinical and molecular features to predict patient outcomes. These models might help identify high‐risk individuals who might benefit from more aggressive therapy, or they can serve as a roadmap for developing—individualized treatment plans. However, several challenges are associated with using machine learning for tumour prognosis. One issue is the lack of high‐quality data sets, which can limit the accuracy and generalizability of the models. Additionally, the complexity of tumour biology and the heterogeneity of tumour samples can make it difficult to identify relevant features for model construction.

Various machine learning methods are used to build prognostic models for tumours, but some issues remain to be addressed. We combined 10 machine learning algorithms into 101 different algorithmic configurations. These encompass elastic net (Enet),^15^, ^16^ supervised principal components (SuperPC), survival support vector machines (survival‐SVM), CoxBoost t,^17^, ^18^ Ridge,^19^ Cox partial least squares regression (plsRcox), generalized boosted regression models (GBM), stepwise Cox,^20^, ^21^ Lasso^22^ and random survival forest (RSF).^23^, ^24^ A leave‐one cross‐validation (LOOCV) framework was utilized to construct prediction models based on the train cohort utilizing 101 algorithm combinations. The models were compared and analysed in seven validation data sets (GSE26193,^25^ GSE26712,^26^ GSE30161,^27^ GSE63885,^28^ GSE9891,^29^ GSE140082^30^ and ICGC_OV_AU). Additionally, we collected 32 published ovarian cancer prognostic models (including mRNA and lncRNA prognostic models). For each prognostic model and all data sets, we performed univariate Cox regression analysis and calculated the C‐index, ultimately demonstrating that the antigen presentation process should be considered a core criterion for evaluating ovarian cancer prognosis.

## 2 METHOD

### 2.1 Acquisition and processing of transcriptome data

Transcriptomic data and corresponding clinical information for ovarian cancer were downloaded from the TCGA database. Using data sets with a sample size greater than 50 in the ICGC database, including OV_AU (*n* = 93) as the validation group for RNAseq to check the model's stability and precision, all data were converted to TPM format, and log2 was converted for subsequent analysis. At the same time, data sets with more than 50 samples in the GEO database are used. GSE26193 (*n* = 107),^25^ GSE26712 (*n* = 185),^26^ GSE30161 (*n* = 58),^27^ GSE63885 (*n* = 75),^28^ GSE9891 (*n* = 278)^29^ and GSE140082 (*n* = 380^30^) ovarian cancer chip data were included as validation sets. The normalizeBetweenArrays function in the limma package is used for data correction of the chip data. The immunotherapy data are based on IMvigor210 (plus TIDE Online prediction data) and comes from the R package IMvigor210CoreBiologies.^31^, ^32^ Samples with missing information were excluded, and in cases where a gene had multiple rows in the expression matrix, the data in those rows were averaged.

### 2.2 Acquisition and processing of single‐cell SEQ data

The single‐cell data set, which included 5 normal and 7 tumour samples for 12 samples, was extracted from GSE184880^33^ in the GEO database. We used R software (version 4.1.3) and the Seurat analytic tool for data analysis. Under the condition of cell quality control, the mitochondrial content should be less than 40%, for the reason that after cell disruption, there may be an increase in the proportion of mitochondrial or nuclear RNAs, which can impact variance estimation and PCA results. To mitigate the influence of disrupted cells on the experiment, we established a standard criterion that ‘mitochondrial content should be less than 40%’. And the limit interval of UMI count and gene count should be 200–100,000 and 200–10,000, respectively. Data normalization, selection of highly variable genes (2000), data transformation (eliminating cell cycle effects). The argument vars.to.regress = c (‘s.core’, ‘G2M.Score’) uses the functions NormalizeData, FindVariableFeatures and ScaleData from the Seurat package, respectively. Batch effects were processed using harmony. The subsequent use of dimensional reduction methods Uniform Manifold Approximation and Projection (UMAP), t‐Distributed Stochastic Neighbour Embedding **(**t‐SNE) and clustering algorithm Louvian, all from Seurat. With criteria of *p‐*value less than 0.05, log2FC larger than 0.25, and a ratio of expression greater than 0.1, we used the FindAllMarkers function to determine differential genes between clusters or cell types. We annotated distinct cell subtypes based on differentially expressed genes.

### 2.3 Acquisition of antigen presentation‐related genes

By searching for related signatures in the MSigDb database, the following two signatures were selected, with a total of 146 genes^34^: monocle2 REACTOME_ANTIGEN_PRESENTATION_FOLDING_ASSEMBLY_AND_PEPTIDE_LOADING_OF_CLASS_I_MHC, REACTOME_MHC_CLASS_II_ANTIGEN_PRESENTATION MSigDb database link: https://www.gsea‐msigdb.org/gsea/msigdb/human/genesets.jsp. Download all genes included in these two signatures to locally construct a list of antigen presentation‐related genes. If a gene symbol cannot be found in the expression matrix, search the GeneCards website (https://www.genecards.org/) to replace.

### 2.4 Consistent cluster analysis

According to antigen presentation‐related genes, consistent clustering, a resamplum‐based method, was used to identify groups within the train cohort. The ConsensusClusterPlus^35^ package performed this procedure. Then, the CDF curve, the consensus score matrix and the PAC score were used to estimate the ideal cluster number.

### 2.5 Weighted correlation network analysis (WGCNA)

Utilizing the WGCNA software, the train's coexpressed gene network was generated.^36^ The suitable soft threshold is calculated to meet the requirements of a scale‐free network. The topological overlap matrix (TOM) is also created from the weighted adjacency matrix, and the associated dissimilarity (1‐TOM) is generated. The dynamic tree‐cutting method is used for module identification. The gene modules with the strongest association were chosen for additional research to determine the gene modules strongly connected to antigen presentation. Genes linked to antigen presentation were characterized as having both high GS and MM.

### 2.6 Cell annotation analysis

We first used epithelial cell marker (‘EPCAM’, ‘KRT18’, ‘KRT19’, ‘CDH1’); Fibroblast markers (‘COL1A1’, ‘DCN’, ‘COL1A2’, ‘THY1’); Endothelial cell markers (‘FLT1’, ‘RAMP2’, ‘PECAM1’, ‘CLDN5’); T cell markers (‘TRAC’, ‘CD3D’, ‘CD3G’, ‘CD3E’); NK cell markers (‘NCAM1’, ‘NKG7’, ‘KLRD1’, ‘GNLY’); B cell markers (‘IGHM’, ‘IGHA2’, ‘CD79A’, ‘IGHG3’); Neutrophil markers (‘FCGR3B’, ‘CSF3R’, ‘LYZ’, ‘CST3’). On this basis, epithelial cells, immune cells and fibroblasts were isolated and grouped for analysis to explore their tumour heterogeneity, and some charts, such as UMAP, t‐SNE, bar chart and heat map, were made.

### 2.7 Subgroup analysis of each cell group

Seurat's standard process was also used to separate immune cells, epithelial cells and fibroblasts to further distinguish the subgroups, and specific markers were used for the subgroups as markers for the group, and UMAP was shown.

### 2.8 CNV analysis of epithelial cells

We used InferCNV software, using normal epithelial cells as a reference, for CNV analysis of tumour cell subsets, mainly to identify malignant cells among them.

### 2.9 Quasi‐temporal analysis of epithelial cells

We used monocle2 software to perform quasi‐time series analysis for epithelial cell subsets, dimension reduction algorithm using DDRTree, and the rest using default parameters. You get the process of cell differentiation.

### 2.10 Transcription factor analysis and tumour pathway analysis

We analysed transcription factors in different cell subpopulations using the decoupleR software, using dorothea. The feature mentioned above enables the calculation of the activity score at the level of each cell, describing how much each cell is enriched in TF and its downstream targets (regulators). To predict the activity of tumour‐associated pathways, the PROGENY model was used.

### 2.11 Analysis of inter‐cell communication

Using Liana software, the algorithm utilizes ‘connectome’, ‘sca’, ‘logfc’, ‘natmi’ and ‘cellphonedb’ to evaluate cell‐to‐cell communication within each cell type during classification.

### 2.12 Association analysis of tumour cells and bulk data

We used Scissor software to correlate the expression and survival data of the train with single‐cell data and set the alpha value to 0.05 to obtain negative and positive cells associated with generation.

### 2.13 Establishing risk profiles associated with tumours

First, the survival conditions of the train data set were correlated with single‐cell data to identify positive and negative cells related to survival, and then the differential genes of positive and negative cells were calculated (the screening conditions encompassed a log2FC greater than 0.25, *p*‐value less than 0.05, and an expression ratio greater than 0.1). The gene set of risk genes was obtained by the intersection of differential genes and the module genes obtained by WGCNA analysis. A single‐factor Cox analysis was performed to identify tumour‐related genes having prognostic value. Prognostic models were built using 101 machine learning methods. Therefore, the algorithm can provide a risk score for each patient in this way. Based on the group's cutoff value of the group determined by the surv_cutpoint function, patients in the train cohort and other cohorts were divided into groups of high and low risk. We then looked at how the predictions of the two groups changed with each other and assessed the accuracy of the model.

### 2.14 Risk characteristics generated by an integrated approach based on machine learning

We used 101 method combinations with 10 machine learning algorithms to create a model called Antigen presentation‐associated genetic markers (AIDPS) with excellent precision and steady performance. The inclusive set of algorithms comprises elastic network (Enet), stepwise Cox, CoxBoost, Lasso, random survival forest (RSF), supervised principal component (SuperPC), Cox partial least squares regression (plsRcox), generalized enhanced regression model (GBM), survival support vector machine (Survival‐SVM) and Ridge. The procedure for generating signatures is outlined as follows: (a) In the TRAIN‐OV cohort (described in the previous step), prognostic genes are identified using univariate Cox regression; (b) Next, 101 algorithm‐based combinations of prognostic genes are utilized to establish a prediction model through a leave‐one cross‐validation (LOOCV) framework within the TRAIN‐OV cohort; (c) The models are subsequently tested across seven validation data sets (GSE26193, GSE26712, GSE30161, GSE63885, GSE9891, GSE140082 and ICGC_OV_AU); (d) the Harrell consistency index (C‐index) is calculated for each model across all validation data sets, and the model with the highest average C‐index is chosen as the best option.

### 2.15 Comparison with prognostic models in other literature

We conducted univariate Cox regression analyses on the AIDPS model and 32 other previously published models across eight data sets (TCGAOV, ICGCOV_AU, GSE9891, GSE63885, GSE30161, GSE26712, GSE26193 and GSE140082) to observe their impact on prognosis. Heatmaps were generated to visualize the results. Subsequently, we computed the C‐index for all models across the eight data sets and plotted them to compare their diagnostic performance.

### 2.16 Statistical analysis

R 4.1.3 software is used for all data processing, statistical analysis and charting. The Pearson correlation coefficient measures the relationship between two continuous variables. The Wilcoxon rank‐sum or *t*‐test was used to compare continuous variables, whereas the Chi‐squared test was used to compare categorical variables. The best cutoff value may be found using the survminer package. Utilizing survival kits, Cox regression and Kaplan–Meier analysis were carried out. The CompareC package can be used to compare c‐indices for various variables.

## 3 RESULTS

### 3.1 Single‐cell expression profile of ovarian cancer

**FIGURE 1:**
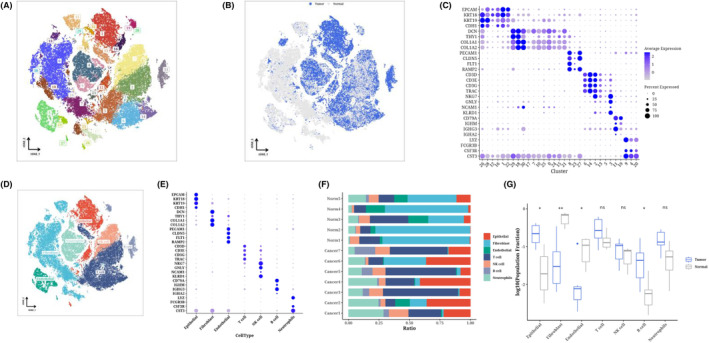
Single‐cell classification of ovarian cancer. t‐SNE plots of ovarian cancer single‐cell data classified by cluster, cell source and cell type (A, B, D). Bubble diagram (C) of the expression of each marker in each cluster. Bubble diagram of the expression of each marker in each cell type (E). The cell types of each sample form a bar chart (F from top to bottom, with the last seven tumour samples and the first five paracancer/normal samples). Boxplot of cell composition of tumour and paracancer group (G). *p < 0.05; **p < 0.01.

After performing dimensionality reduction cluster analysis on the single‐cell data, a total of 31 clusters were obtained (Figure 1A). We observed the distribution of cells from each sample source on the t‐SNE diagram (Figure 1B), using epithelial cell markers (‘KRT19’, ‘CDH1’, ‘KRT18’, ‘EPCAM’); fibroblast marker (‘COL1A2’, ‘THY1’, ‘COL1A1’, ‘DCN’); endothelial cell marker (‘CLDN5’, ‘PECAM1’, ‘RAMP2’, ‘FLT1’); T‐cell marker (‘CD3E’, ‘CD3D’, ‘TRAC’, ‘CD3G’); NK cell maker (‘KLRD1’, ‘NKG7’, ‘NCAM1’, ‘GNLY’); B cell marker (‘IGHA2’, ‘CD79A’, ‘IGHG3’, ‘IGHM’); Neutrophil marker (‘LYZ’, ‘FCGR3B’, ‘CSF3R’, ‘CST3’) was used to perform bubble maps for each subgroup (Figure 1C). According to the expression of marker genes, each cluster was annotated as each cell type (Figure 1D). The bubble diagram shows the marker expression of each cell type, confirming the correctness of the cell type (Figure 1E). The normal samples contained more fibroblasts and endothelial cells than the tumour samples. The tumour sample contained higher epithelial and B cells (Figure 1F,G).

### 3.2 Subclassification analysis and cell trajectory analysis of epithelial cells

**FIGURE 2:**
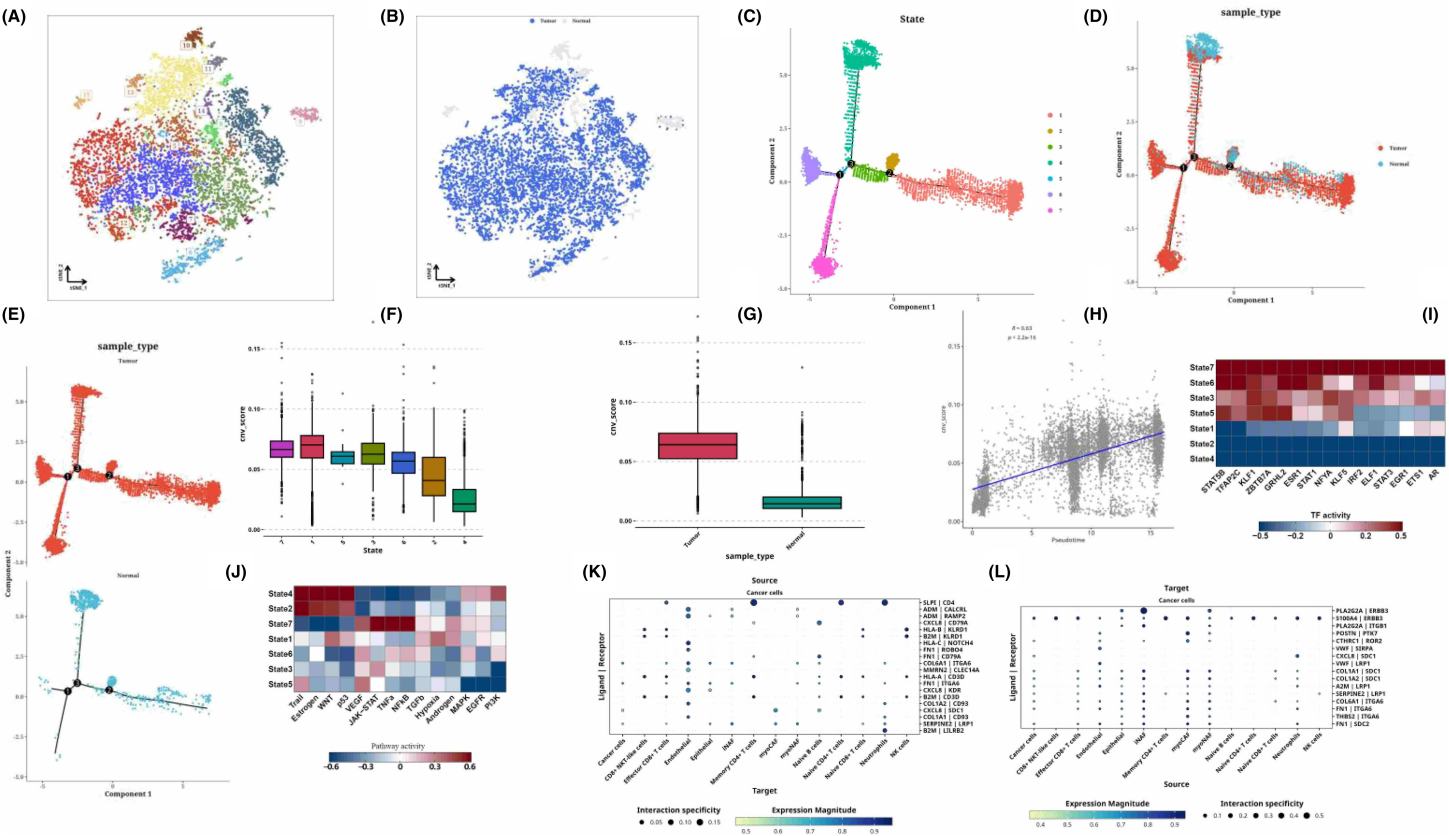
Results of subclassification analysis and cell trajectory analysis of epithelial cells. Epithelial cell data are classified by cluster and colour‐coded t‐SNE plots (A, B). monocle2 locus maps based on epithelial cell data, coloured by state and cell source, respectively (C–E). Box plot (F, G) of CNV score by state and cell source. Scatter plot of correlation between simulated time and CNV score (H). Heat maps of transcription factor activity by state (I). Heat maps of relevant pathways for each State (J). Cell communication bubble map (K, L) with tumour cells as source and target. *p < 0.05; **p < 0.01.

The epithelial cells were isolated for dimensional reduction cluster analysis. The t‐SNE diagram displayed 16 clusters along with the sources of their cell samples. (Figure 2A,B). We performed cell locus analysis on the epithelial cells, which showed the locus correlation among cell sources. At the same time, it can be seen that State4 (normal cells) may be the starting point of tumour analysis and differentiation, bifurcating into three types in the direction of State1, State7 or State6 (Figure 2C–E). We also performed CNV analysis on cells in each State and found that the CNVscore of 7 states was State7&gt. State1> State5> State3> State6> State2> State4, and cancer. Normal, indicating that State4 is correct as the starting point. Pearson correlation analysis was conducted on CNVscore and pseudo time later, and the two groups were positively correlated (*R* = 0.63, *p* < 2.2e‐16) (Figure 2F–H). Through transcription factor analysis, we can see that State7, 6, 5, 3, representing tumours, is enriched in STAT5B, TFAP2C, KLF1, ZBTB7A, etc. (Figure 2I). From the perspective of pathway analysis, State4 is related to p53 and PI3K, and State7 is related to NFkB and TNFa (Figure 2J). Then, using a variety of cell types, we conducted a cell communication analysis, focusing on how tumour cells interact with other types of cells. We specifically showed the source and target locations on the communication bubble map of tumour cells (Figure 2K,L).

### 3.3 Analysis of subclassification of immune and fibroblasts

**FIGURE 3:**
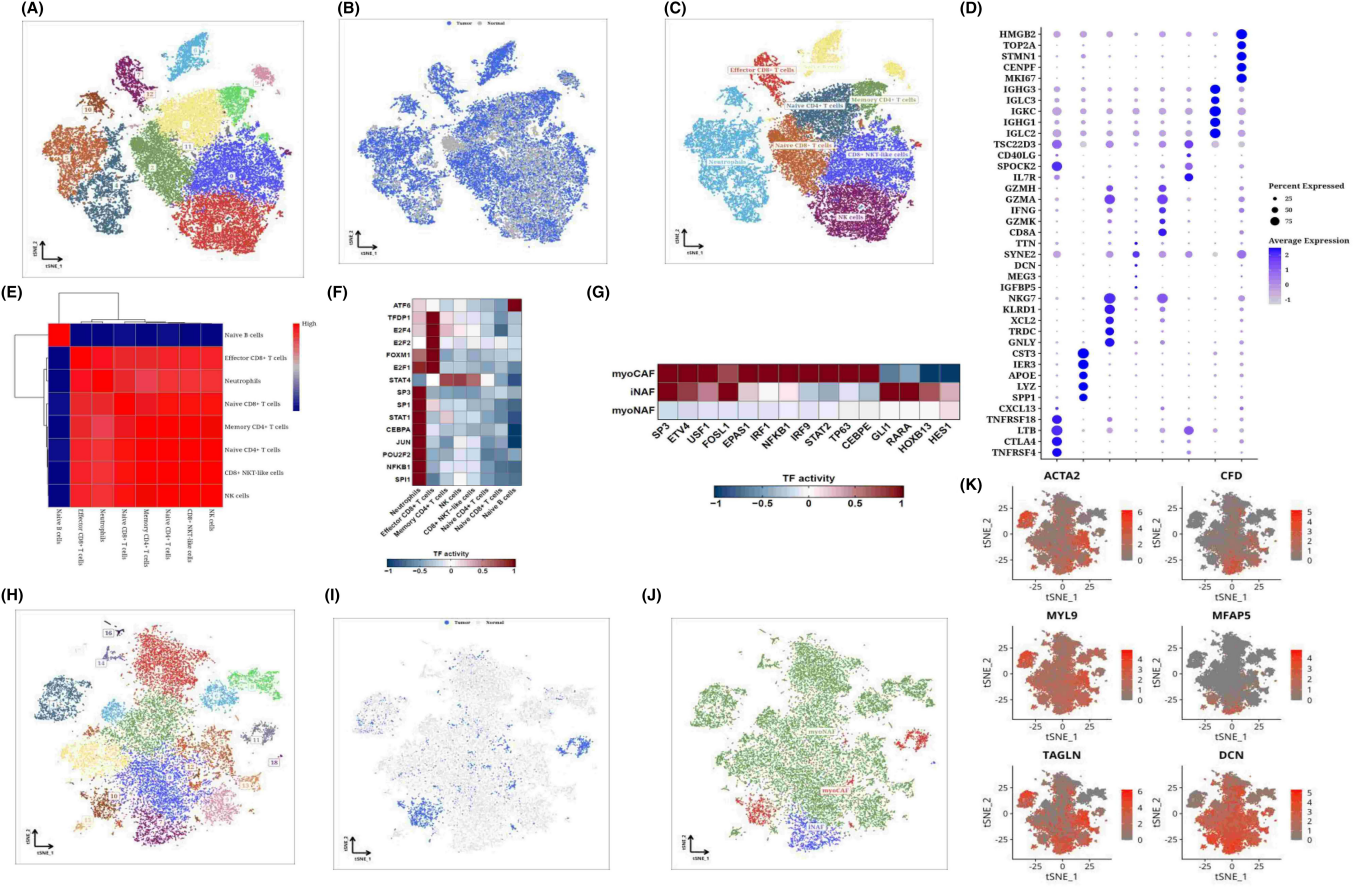
Analysis results of the subclassification of immune and fibroblasts. Immune cell data are classified by cluster and colour‐coded t‐SNE plots (A, B). t‐SNE diagram (C) of cell annotation results after SCType software annotation. Bubble map of marker expression by cell type (D). Pearson correlation heat maps of immune cells by cell type (E). Heat maps of transcription factor activity by immune cell type (F). Heat maps of transcription factor activity in myoNAF, myoCAF and iNAF cells (G). Fibroblast data were classified by cluster and colour‐coded t‐SNE plots (H, I). t‐SNE diagram of fibroblast annotation results using a specific marker (J) after expression annotation. Expression map of t‐SNE of specific marker (‘ACTA2’, ‘CFD’, ‘MYL9’, ‘MFAP5’, ‘TAGLN’, ‘DCN’) (K).

The immune cells were isolated for dimensional reduction cluster analysis. 13 clusters were shown on the t‐SNE diagram (Figure 3A), and the sample source of the cells could be seen from the t‐SNE diagram (Figure 3B). SCType software was used to annotate the cells and obtain the t‐SNE diagram of the annotated results (Figure 3C). The expression of marker genes in each cell type is shown by the bubble map (Figure 3D). Pearson algorithm was used for correlation analysis of cell types, and it was observed that Naive B cells were less similar to other cell groups, while other cell groups were more similar (Figure 3E). We performed transcription factor analysis on Naive B cells and found that Naive B cells are enriched in ATF6, neutrophils are enriched in NFKB1 and SPI1, and Effector CD8+ T cells are enriched in TFDP1, E2F4 and E2F2. STAT4 was enriched in Memory CD4+ T cells, NK cells, and CD8+ NKT‐like cells (Figure 3F). Subsequently, we isolated the fibroblasts and performed dimensional reduction cluster analysis separately. The t‐SNE diagram showed 19 clusters (Figure 3H), from which we could see the sample source of the cells (Figure 3I). We can classify fibroblasts into myofibroblasts or inflammatory phenotypes (Figure 3J). Group 7,18 expressed the characteristics of inflammatory subtypes, such as CFD, MFAP5 and DCN, and because most of its cells were derived from normal samples, it was named iNAF (Figure 3K). The remaining subgroups were identified as myofibroblasts because of upregulated myofibroblast markers, including α smooth muscle actin (αSMA, also known as ACTA2) and contractile proteins (TAGLN, MYLK, MYL9), and because of differences in their cell origin, divided into two categories, with subgroups 10,11 being myoCAF and 11 being MYOCAF. The remaining clusters are myoNAF (Figure 3K). The distribution of the three CAFs was shown on the t‐SNE diagram (Figure 3J), and the different transcription factors enriched by the three CAFs could be seen from the analysis of transcription factors (Figure 3G).

### 3.4 Association analysis of single cells and bulk data and acquisition of antigen‐presenting related prognostic gene sets

**FIGURE 4:**
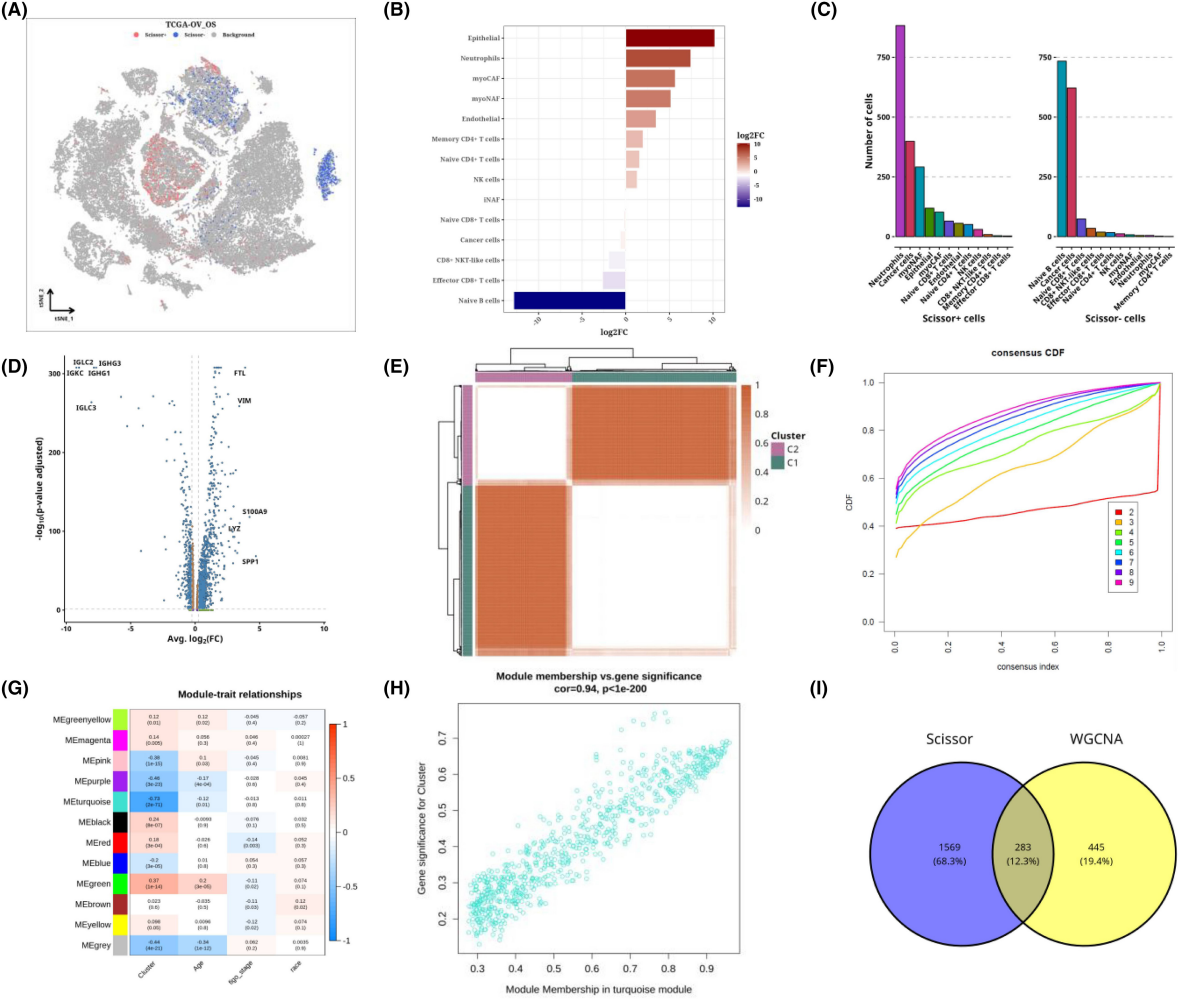
Association analysis of single cells with bulk data and acquisition of antigen presentation‐related prognostic gene set results. Distribution of negative and positive cells in ovarian cancer single‐cell data t‐SNE diagram (A). The number of positive versus negative cells in each cell type is shown in a bar chart (B). Histogram (C) of the number of positive and negative cells by cell type. Volcanic map of differential gene analysis of positive and negative cells (D). Heat map of conformance analysis results for 146 antigen‐presenting genes in TRAIN data (E). Consistency CDF curve for each K value (F). Heat map of clinical variables such cluster, age, tumour stage and race and their link with the WGCNA module (G). Scatter plot (H) of correlation between gene significance (GS) and module member (MM) in the turquoise module. Winn diagram (I) of Scissor differential genes and WGCNA screened genes.

We used Scissor software to correlate TRAIN‐OV's expression and survival data with single‐cell data and obtained 2013 positive and 1534 negative cells. Where positive cells indicate that they may have a greater impact on survival than negative cells, we use t‐SNE diagrams to show the classification of positive, negative and background cells (Figure 4A). Comparing the number of positive and negative cells in each cell type, we found that epithelial cells and neutrophils had a larger proportion of positive cells, while Naive B cells had a larger proportion of negative cells (Figure 4B). After analysing the composition of positive and negative cells, we can see that many positive cells exist in neutrophils and cancer cells, and many negative cells exist in Naive B and cancer cells (Figure 4C). Differential genetic analysis of positive and negative cells shows the difference and distribution results, presented as a volcano map (Figure 4D). We performed a consistent cluster analysis based on the expression of 146 genes associated with antigen presentation, in which all ovarian cancer samples were initially grouped into k (*k* = 2–9) clusters. The ratio of the cumulative distribution function (CDF) curve of the consistency score matrix and the fuzzy clustering (PAC) statistic indicates that the best number is obtained when *k* = 2, resulting in two cluster clusters (C1 and C2) (Figure 4E,F). The weighted correlation network analysis (WGCNA) process, the association between the modules and clinical variables such as immunological cluster, age, tumour stage and race was also calculated. The turquoise module demonstrated the highest correlation with the cluster module‐trait relationship (Figure 4G). The turquoise module's gene significance (GS) and module members (MM) were compared, and the correlation coefficient was 0.94, demonstrating superior gene module construction (Figure 4H). 283 overlapped genes were extracted for further study by intersection with the Scissor differential gene results (Figure 4I).

### 3.5 Construction of risk model generated by machine learning‐based integrated approach (AIDPS)

**FIGURE 5:**
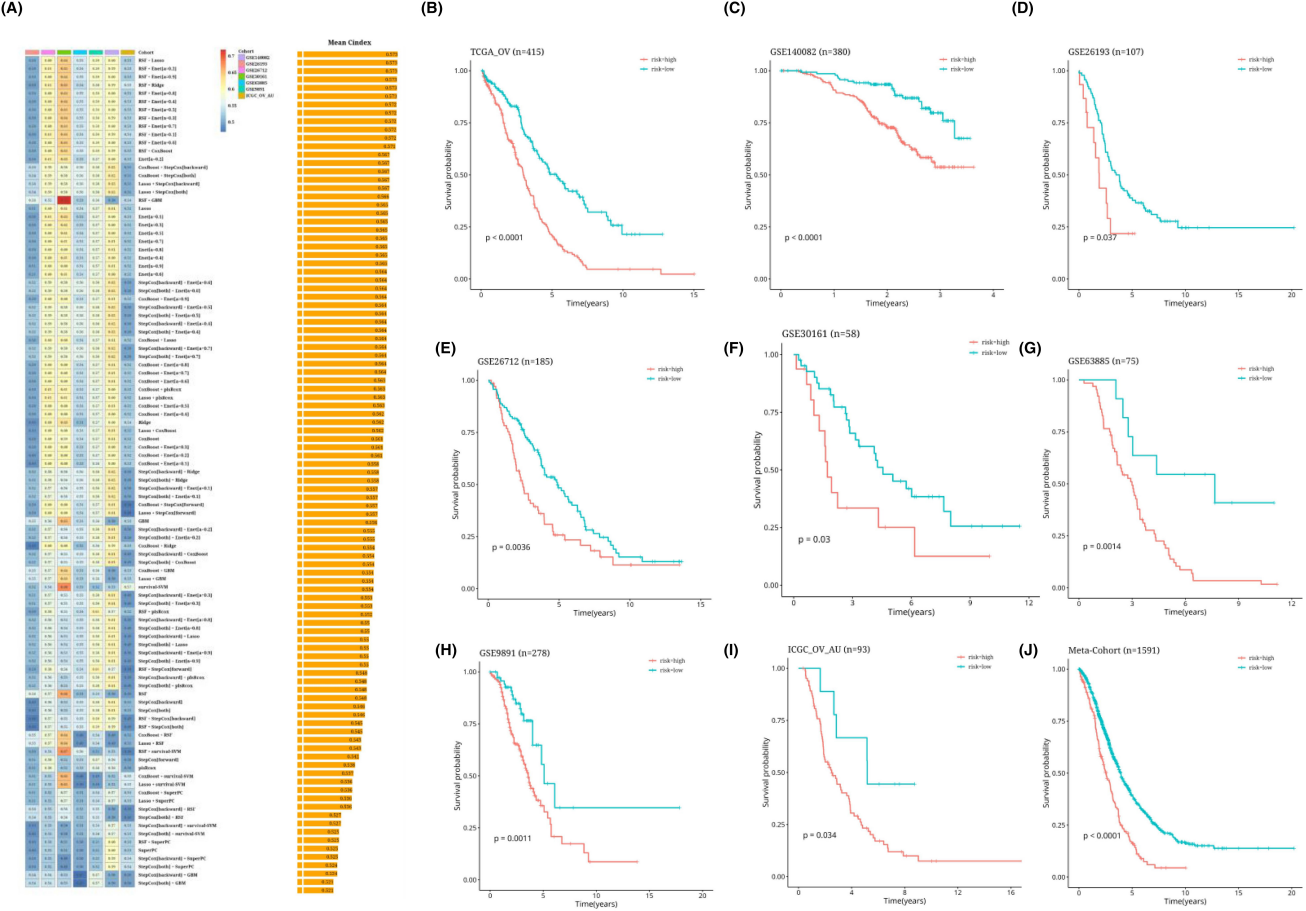
Risk model construction results generated by machine learning‐based integrated approach (AIDPS). Heat maps and average C‐index bars (A) of seven validated data sets in 101 machine learning prognostic models. Survival analysis results of TRAIN training data and seven validation data sets (B–I). Merge survival analysis results (J) for all data sets.

Based on the expression profiles of 283 genes associated with antigen presentation and the intersection of genes using the data, 22 prognostic genes were identified by univariate Cox analysis (*p* < 0.05). These 22 genes underwent our machine learning‐based integration programme to develop consensus AIDPS. We fit 101 prediction models to the TRAIN‐OV data set using the LOOCV framework and then compute the C‐index for each model across all validation data sets (Figure 5A). Interestingly, the best model was RSF + Lasso, having the highest average C‐index (0.573), and this combined model led all validated data sets in C‐index (Figure 5A). The risk score was then calculated for each patient using the prognostic gene expression that was optimally represented in the model, and all patients were divided into two groups based on the optimal threshold established by the survminer package: the high‐risk group and the low‐risk group, as depicted in the figure. In Figure 5B–I, in the TRAIN‐OV data set and the other seven validated data sets, compared with patients in the low‐risk group, those in the high‐risk group had significantly poorer overall survival (OS) (all *p* < 0.05). Meta‐cohorts that combine all samples show the same trend (*p* < 0.05) (Figure 5J).

### 3.6 Evaluation of AIDPS model

**FIGURE 6:**
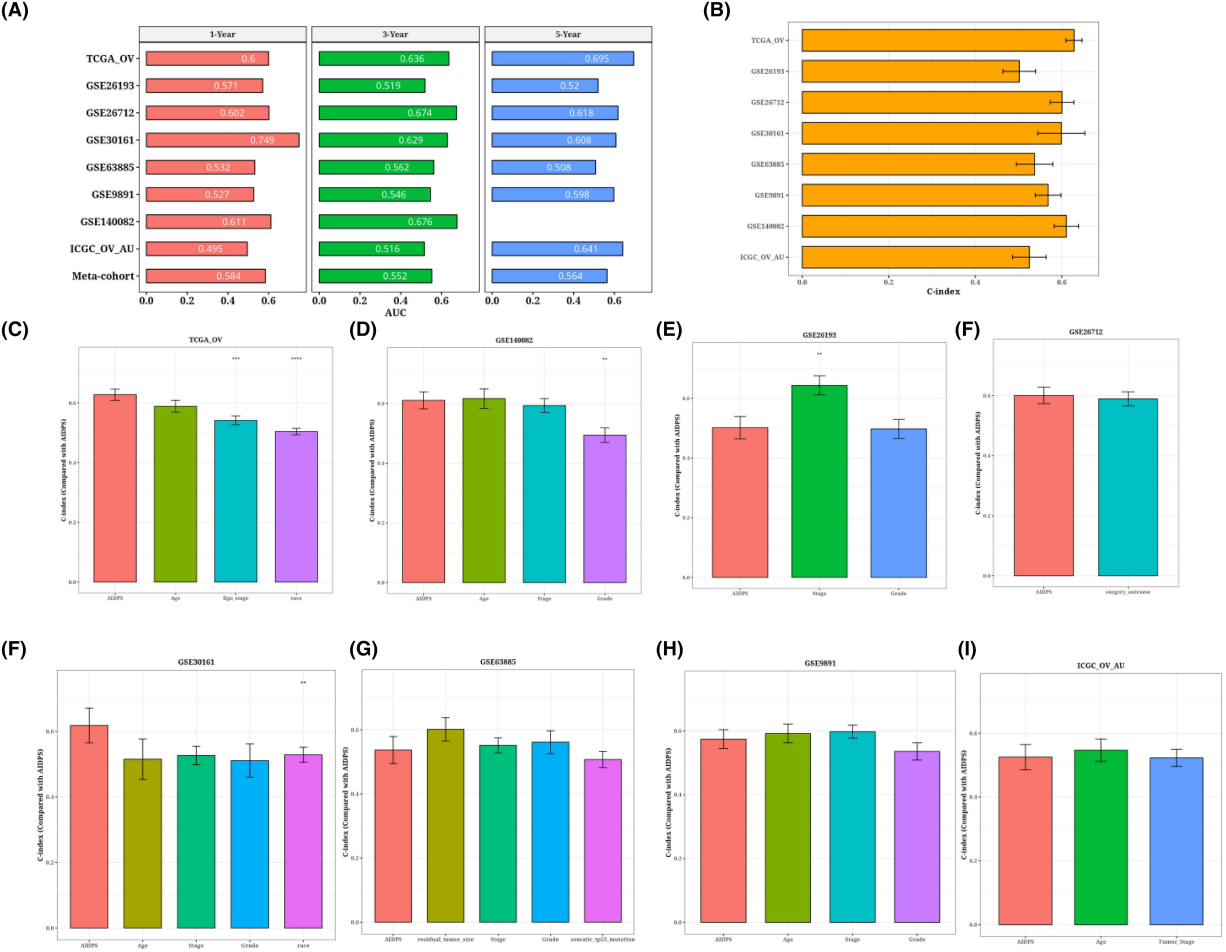
Results of evaluation and analysis of the AIDPS model. Bar chart of AUC values for each data set at 1, 3 and 5 years (A). C‐index error histogram (B) for each data set. C‐index error histogram (C–I) of AIDPS and other clinical measures for TRAIN training set and 7 validation data sets. **p < 0.01; ***p < 0.001; ****p < 0.0001.

ROC analysis measured the discrimination of AIDPS, and the 1‐year, 3‐year and 5‐year AUC in TRAIN‐OV were 0.6, 0.636 and 0.695, respectively. The AUC of GSE26193 was 0.571, 0.519 and 0.52. In GSE26712, the values are 0.602, 0.674 and 0.618. 0.749, 0.629 and 0.608 in GSE30161; the values of GSE63885 are 0.532, 0.562 and 0.508. 0.527, 0.546 and 0.598 in GSE9891; 0.611 and 0.676 in GSE140082; in ICGC_OV_AU, the values are 0.495, 0.516 and 0.641. They are 0.584, 0.552 and 0.564 (Figure 6A). We present a bar chart of the C‐index [95% confidence interval] for each cohort (Figure 6B). These indicators show that AIDPS performs consistently well across a number of independent cohorts. According to a prior study, clinical features, including the stage of the tumour, are also utilized to determine the prognosis of ovarian cancer in clinical practice. Consequently, we compared AIDPS performance with prognostications from different clinics. As shown in Figure 6C–I. The accuracy of AIDPS was significantly better than other variables, including age and Stage stage.

### 3.7 Comparison with prognostic models in other literature

**FIGURE 7:**
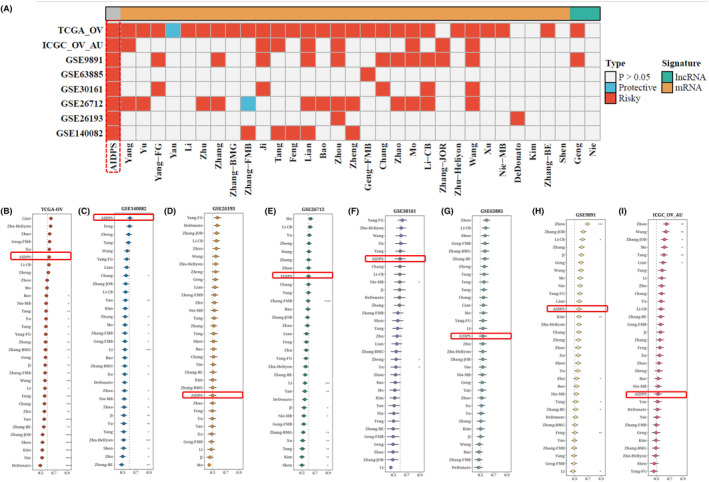
Results of comparison with the upper prognostic models in other literature. Prognostic correlation heat maps of 32 other models reported in the literature plus models in eight data sets (A). C‐index error point plots (B–I) for each model in the TRAIN training set and seven validation data sets. *p < 0.05; **p < 0.01; ***p < 0.001; ****p < 0.0001.

With the advancement of next‐generation sequencing and big data technology in recent years, many prognostic and predictive gene expression profiles based on machine learning have been developed. To compare the performance of AIDPS with other prognostic models, we conducted a comprehensive search of published prognostic models in the literature. The severe lack of miRNA data in some chips' validation data sets made using a miRNA prognostic model impossible. Finally, 32 prognostic models (including mRNA and lncRNA prognostic models) were collected. These characteristics are linked to a variety of biological processes, including immunological response, autophagy, iron death, dryness, epithelio‐mesenchymal transition, copper death, lipogenesis, N6‐methyladenosine, epigenetics, ageing, WNT, glycolysis, vitamin D, drug sensitivity and hypoxia. We ran univariate Cox regression analysis on each prognostic model and all data sets and found that only our model was significantly linked with prognostic outcomes in nearly all cohorts (Figure 7A), indicating the stability of AIDPS. Furthermore, the AIDPS C‐index was compared to other parameters. Notably, AIDPS outperformed most models in each data set (Figure 7B–I). On their training data sets and some external data sets, most models exhibit good performance (e.g., Xu, Zhu − Heliyon) but poorly on others (Figure 7A). This could be due to the resulting model's poor generalization due to overfitting. Multiple machine learning methods have reduced the dimensionality of our model, giving it greater extrapolation potential.

### 3.8 Predicted response to immunotherapy/chemotherapy in the risk group

**FIGURE 8:**
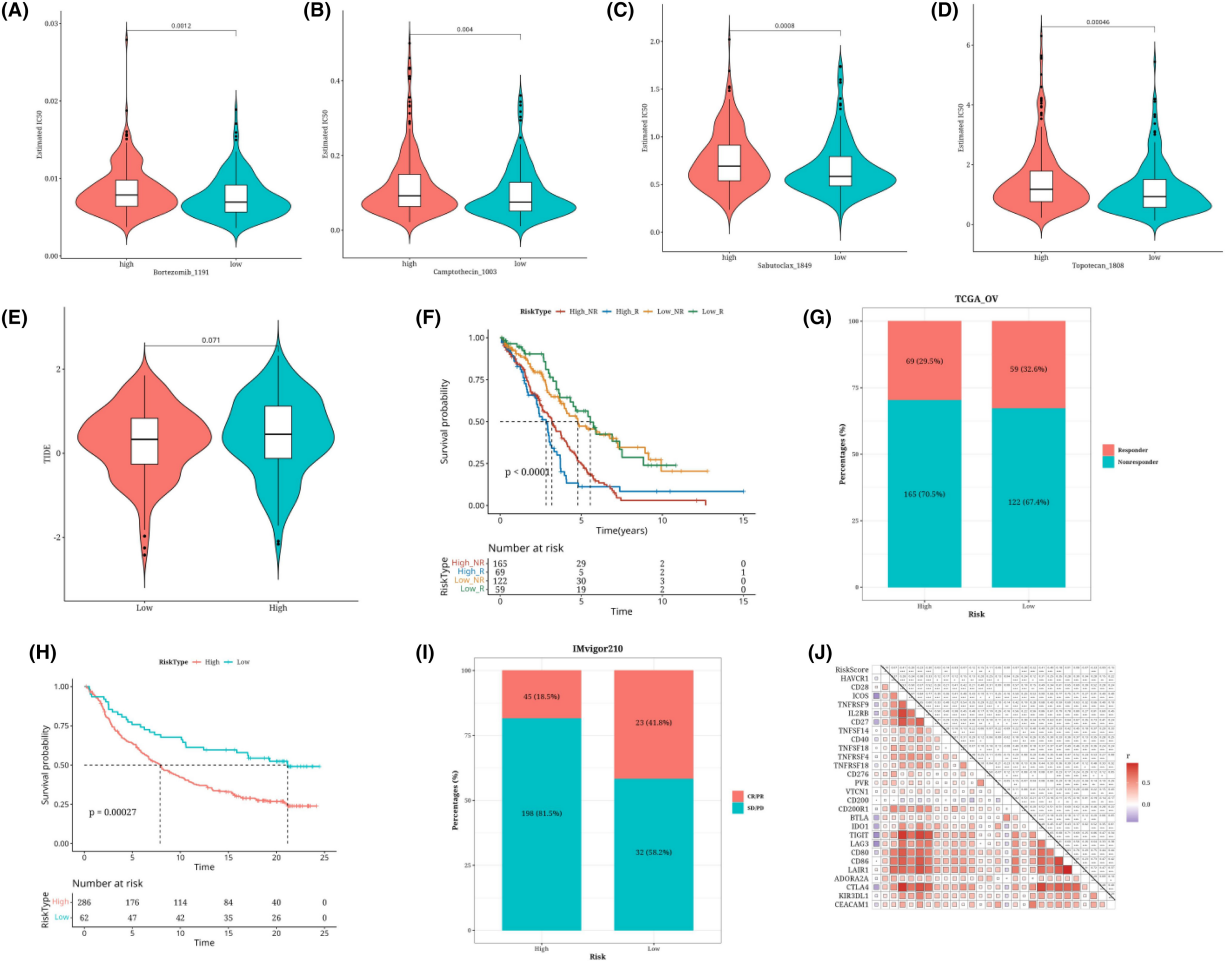
Predicted response analysis results of immunotherapy/chemotherapy in the risk group. The difference in IC50 values of chemotherapeutic drugs Bortezomib_1191, Camptothecin_1003, Sabutoclax_1849 and Topotecan_1808 in high‐ and low‐risk groups violin chart (A–D). Train data for TIDE values differences in risk groups Violin plot (E). Survival analysis results for the risk group plus immune response (F). The percentage of immunological responses in the risk group is shown in a bar chart (G). Survival analysis results of the IMvigor210 data set using the RSF + Lasso prognostic model (H). Bar chart (I) of the proportion of immune responses in the risk group in the IMvigor210 data set. Risk value and the expression of immune checkpoint genes are correlated in a heat map (J).

We found significant differences in the IC50 values of Sabutoclax_1849, Camptothecin_1003, Topotecan_1808 and Bortezomib_1191 among the high and low‐risk groups (Figure 8A–D). The two risk groups showed a nearly significant difference in TIDE values according to the online analysis of TIDE (*p* = 0.071, Figure 8E). According to survival analyses, the high‐risk response group's prognosis was poorer (Figure 8F). The bar chart shows the proportion of responders and non‐responders in the risk group (Figure 8G). Using immunotherapy data IMvigor210, risk value estimates were made using prognosis, and a survival analysis curve was plotted, which found that the prognosis for the high‐risk group was considerably poorer than that of the low‐risk group (Figure 8H). The bar chart shows the proportion of responders and non‐responders in the risk group in the IMvigor210 data set (Figure 8I). In the analysis of immune checkpoints, risk scores and some immunological checkpoints exhibit a strong association (Figure 8J).

## 4 DISCUSSION

The AJCC staging system^37^ for ovarian cancer, as a traditional approach to clinical management, has some value in treatment decision‐making and monitoring strategies, but due to heterogeneous clinical results at the same time, its capability is limited. This insufficient method can result in probable overtreatment or undertreatment. Due to advancements in molecular biology and immunology, treatments for ovarian cancer have changed to include immune checkpoint inhibitor (ICI) therapy and anti‐angiogenic medicines such as beizumab. The existence of several treatment alternatives necessitates the development of improved individualized assessment tools for patients in order to facilitate the implementation of clinical decisions. However, there is currently a lack of reliable prognostic biomarkers to identify ‘high‐risk’ ovarian cancer patients who may benefit from treatment with Pembrolizumab and ICI. We investigated the relationships between antigen presentation profiles and prognosis, recurrence, and therapeutic benefit to fill this knowledge gap and provide better‐personalized treatment strategies for ovarian cancer patients.

Specifically, a variety of machine learning methods and algorithm combinations are used by us, including elastic networks (Enet), Lasso, random survival forests (RSF), stepwise Cox, Ridge, CoxBoost, supervised principal component analysis (SuperPC), Cox partial least squares regression (plsRcox), generalized enhanced regression models (GBM) and survival support vector machines (survival‐SVM). The elastic net is frequently employed in data sets characterized by high‐dimensional features and demonstrates robust performance in the presence of multicollinearity, where a high degree of correlation exists among the features. Cox Boost is commonly employed in survival analysis, facilitating the construction of more precise survival prediction models. Lasso regression is frequently utilized for feature selection and sparse handling of noisy data. Random Survival Forests (RSF) can be employed to predict the occurrence probability of survival time or events, making it suitable for modelling and forecasting survival data. plsRcox is applicable for the modelling and prediction of survival data, particularly well‐suited for high‐dimensional data sets. Using the LOOCV framework, we construct a prediction model for each algorithm combination based on the TRAIN‐OV queue. We then compared and analysed these models with seven validation data sets from GSE26193, GSE26712, GSE30161, GSE63885, GSE9891, GSE140082 and ICGC_OV_AU. In addition, we collected 32 published ovarian cancer prognostic models (including mRNA and mirRNA prognostic models) and performed univariate Cox regression analyses on all data sets to calculate the C‐index, ultimately suggesting that the antigen presentation process should be considered as the core criterion for evaluating ovarian cancer prognosis.

The AIDPS model quantitatively evaluates the expression levels of antigen presentation‐related genes in patients, and based on this model, we conducted a series of immune‐related analyses. Within the TIDE scoring system, there was no significant difference in immune activity between the two risk groups. We hypothesize that within the tumour microenvironment, antigen presentation‐related genes may have already exerted their influence, but could be hindered by other pathways, leading to suppression of tumour immune cytotoxicity. The results from two databases indicate that patients in the low‐risk group are more likely to benefit from immunotherapy. Additionally, the AIDPS model demonstrates a significant negative correlation with immune checkpoint genes such as ICOS, BTLA, IDO1, TIGIT, LAG3 and CTLA4. Based on this information, we speculate that antigen presentation‐related genes may be influenced by certain factors within the tumour microenvironment, rendering them unable to activate tumour immunity, while immune checkpoint genes may exacerbate the tumour immune suppression state. Studies have shown that ovarian cancer cells develop various strategies to induce immune tolerance. These include secreting multiple immune inhibitory molecules to impair cytotoxic cells, recruiting regulatory cells, altering antigen presentation and effectively evading immune responses.^38^ Furthermore, acquired chemotherapy resistance in ovarian cancer cells may confer resistance to immune checkpoint inhibitor therapy by downregulating antigen presentation mechanisms.^39^ Thus, combination therapy targeting both antigen presentation genes and immune checkpoint genes may improve immune suppression effects and overall treatment outcomes. Our perspective provides new insights for future therapeutic target development, necessitating further experimental research to validate our hypotheses. The immune system is well‐known for its role in surveilling and suppressing the development of malignant tumours by recognizing and attacking tumour cells. T cells play a crucial role in immune surveillance by identifying tumour‐associated antigens presented on the surface of cancer cells. In the case of ovarian cancer, research indicates that Mucin‐16 (MUC16) and Mesothelin (MSLN) are significant tumour antigens, presented respectively by human leukocyte antigen‐I and human leukocyte antigen‐II molecules.^40^ Therefore, we postulate that the pivotal role of antigen presentation in ovarian cancer lies in the recognition of tumour‐associated antigens and their presentation to T cells within the immune system. By studying the relationship between antigen presentation profiles and prognosis, recurrence and drug benefit, we hope to provide better‐personalized treatment strategies for ovarian cancer patients, improve clinical management outcomes and survival and quality of life. In future studies, we can further explore other biomarkers and treatment strategies to lead to more treatment options and better prognosis assessment for ovarian cancer patients.

We conducted single‐cell sequencing analysis on ovarian cancer samples, providing insights into the disease. Firstly, the single‐cell data were subjected to dimensionality reduction and clustering, resulting in 31 clusters annotated with cell types. Cell trajectory analysis revealed a possible tumour origin state (State4) and differentiation trajectories towards various tumour subtypes. Subsequently, the study conducted a subclassification analysis on epithelial cells, identifying seven subgroups with distinct transcription factor activities and pathway features. Furthermore, immune cells and fibroblasts were also classified and functionally annotated. Next, the single‐cell data correlated with bulk data, resulting in 2013 positive and 1534 negative cells. Positive cells were found to be potentially associated with higher survival rates, while negative cells were linked to lower survival rates. Consistency analysis identified two gene modules related to antigen presentation. Finally, a risk model (AIDPS) was developed using machine learning ensemble methods (RSF + LASSO) to predict prognosis in ovarian cancer patients. The AIDPS model demonstrated stable and robust performance in various independent cohorts, outperforming other clinical indicators such as age and tumour stage in accuracy.

T, N, M and AJCC staging are standard instruments for assessing clinical results and guiding treatment choices. Furthermore, whether to employ ACT and emerging biomarkers like TMB, part, microsatellite status and BRCA mutations^41^, ^42^ is also significantly linked to clinical strategies and outcomes. Notably, our antigen presentation assessment system was independent of these factors and was significantly better at predicting prognosis based on the C‐index assessment than other factors. Furthermore, we found 32 published signatures with different functional gene combinations. Few of these characteristics have been properly validated, and even fewer were incorporated into clinical practice. For example, univariate Cox regression revealed that all cohorts other than AIDPS had no characteristics that were maintained to be significant prognosticators. By comparing the predictive advantages of these features, AIDPS also outperformed almost all models on each data set. We note that most models perform well on their training data sets and some external data sets but poorly on others. This could result from the model's poor generalization brought on by overfitting. Due to the dimensioning of our signature using two machine learning methods, extrapolation is more likely. To further verify the clinical solution of AIDPS, AIDPS has demonstrated promising predictive performance in various other diseases.^43^ Furthermore, the relationship between AIDPS and chemotherapy efficacy remains unclear and warrants further investigation. We hypothesize that AIDPS may exhibit a complementary interaction with chemotherapy, thereby enhancing treatment comprehensiveness and improving patient prognosis. Therefore, our AIDPS may be a promising alternative for assessing ovarian cancer prognosis in a clinical setting.

The AIDPS model has much potential for clinical translation and use because it can be replicated using a straightforward PCR‐based assay. In clinical practice, pertinent information can be gathered and sequenced during patient examinations to observe relevant gene expression levels. Prognostic risk models can be constructed based on established formulas to predict and assess patient survival outcomes. Concurrently, predictive results can be utilized to assess patient responsiveness to immunotherapy or immunotherapeutic agents, thereby determining the suitability of current treatment regimens for patient prognosis and guiding the development of further treatment strategies.

Despite being positive, the therapeutic importance of AIDPS in ovarian cancer has a number of drawbacks that should be acknowledged. Firstly, as this study only used retrospective samples, a prospective multicentre cohort should be used in the future to validate AIDPS. This may lead to potential biases in the data set utilized, such as differences in sample sources, sample sizes, and data quality, which could impact the stability and generalization capability of the model. Second, some clinical and biochemical characteristics missing from publicly available data sets may have concealed relationships between IRLS and particular variables. While we employed rigorous cross‐validation methods to assess the performance of the model and conducted validation across multiple data sets, additional independent data sets are still necessary to validate the reproducibility of the model. Thirdly, more in vivo and in vitro research is required to explain the involvement of most of the antigen‐presenting genes from AIDPS in ovarian cancer. Despite proposing a prognostic assessment method based on single‐cell RNA sequencing data and achieving some success, translating these research findings into clinical applications still presents numerous challenges. For instance, further validation of the model's effectiveness and stability in clinical samples is required, along with investigating how to integrate the model into clinical decision‐making and guide treatment strategies. These issues necessitate further research and exploration.

In conclusion, this study provides a comprehensive understanding of ovarian cancer through single‐cell sequencing analysis, uncovering the tumour origin state and differentiation trajectories. This research offers new predictive indicators for immunotherapy and chemotherapy in ovarian cancer. The development of the AIDPS risk model provides a powerful tool for prognosis assessment in ovarian cancer patients, guiding clinical treatment decisions.

## AUTHOR CONTRIBUTIONS

**Wenhuizi Sun:** Data curation (equal); funding acquisition (equal); writing – review and editing (equal). **Ping Xu:** Conceptualization (equal); methodology (equal). **Kefei Gao:** Funding acquisition (lead); project administration (lead); writing – original draft (lead). **Wenqin Lian:** Conceptualization (lead); writing – original draft (equal). **Xiang Sun:** Investigation (lead); visualization (lead); writing – review and editing (lead).

## CONFLICT OF INTEREST STATEMENT

The authors confirm that there are no commercial or financial relationships that could be perceived as a potential conflict of interest with the research conducted.

## Data Availability

The data sets presented in this study can be found in online repositories and could be required on corresponding authors.
